# Adaptive and Innate Immune Cells in Fetal Human Cytomegalovirus-Infected Brains

**DOI:** 10.3390/microorganisms8020176

**Published:** 2020-01-25

**Authors:** Yann Sellier, Florence Marliot, Bettina Bessières, Julien Stirnemann, Ferechte Encha-Razavi, Tiffany Guilleminot, Nacilla Haicheur, Franck Pages, Yves Ville, Marianne Leruez-Ville

**Affiliations:** 1 Service de Gynécologie-Obstétrique, Hôpital Universitaire Necker-Enfants-malades, AP-HP, 149 rue de Sèvres, 75015 Paris, France; yann.sellier@aphp.fr (Y.S.); j.stirnemann@gmail.com (J.S.); 2 EHU 7328 PACT, 75015 Paris, France; marianne.leruez@aphp.fr; 3 Université Paris Descartes, Sorbonne Paris Cité, 75015 Paris, France; florence.marliot-ext@aphp.fr (F.M.); bettina.bessieres@aphp.fr (B.B.); ferechte.razavi@nck.aphp.fr (F.E.-R.); tiffany.guilleminot@aphp.fr (T.G.); franck.pages@aphp.fr (F.P.); 4 INSERM U872, plateforme d’Immuno-monitoring, service d’Immunologie Biologique, Hôpital Européen Georges-Pompidou, AP-HP, 75015 Paris, France; nacilla.haicheur@aphp.fr; 5 Service d’histologie-Embryologie-Cytogénétique Hôpital Universitaire Necker-Enfants-malades, AP-HP, 75015 Paris, France; 6 Institut Imagine, Université Paris Descartes (INSERM U) 1163, 75015 Paris, France; 7 Laboratoire de Virologie, Hôpital Universitaire Necker-Enfants-malades, AP-HP, Centre National de Référence, laboratoire associé Cytomégalovirus, 75015 Paris, France

**Keywords:** cytomegalovirus, fetal brain, PD-1, LAG-3, exhaustion, immune cells

## Abstract

Background: The understanding of the pathogenesis of cytomegalovirus (CMV)-induced fetal brain lesions is limited. We aimed to quantify adaptive and innate immune cells and CMV-infected cells in fetal brains with various degrees of brain damage. Methods: In total, 26 archived embedded fetal brains were studied, of which 21 were CMV-infected and classified in severely affected (*n* = 13) and moderately affected (*n* = 8), and 5 were uninfected controls. The respective magnitude of infected cells, immune cells (CD8^+^, B cells, plasma cells, NK cells, and macrophages), and expression of immune checkpoint receptors (PD-1/PD-L1 and LAG-3) were measured by immunochemistry and quantified by quantitative imaging analysis. Results: Quantities of CD8^+^, plasma cells, NK cells, macrophages, and HCMV^+^ cells and expression of PD-1/PD-L1 and LAG-3 were significantly higher in severely affected than in moderately affected brains (all *p* values < 0.05). A strong link between higher number of stained cells for HCMV/CD8 and PD-1 and severity of brain lesions was found by component analysis. Conclusions: The higher expression of CD8, PD-1, and LAG-3 in severely affected brains could reflect immune exhaustion of cerebral T cells. These exhausted T cells could be ineffective in controlling viral multiplication itself, leading to more severe brain lesions. The study of the functionality of brain leucocytes ex vivo is needed to confirm this hypothesis.

## 1. Introduction

Human Cytomegalovirus (HCMV) congenital infection is the most frequent cause of congenital infections, affecting 0.7% of live births worldwide [1]. It is the leading non-genetic cause of sensorineural hearing loss (SNHL), the major infection-related cause of congenital malformations in high-income countries and a major cause of neurological disability. It accounts for up to 10% of all cases of cerebral palsy [2] and 20–25% of all congenital SNHL [3].

Fetuses experience fetal viremia and viral replication in many organs in the acute phase of infection. This acute phase is constantly followed by a chronic infection, with persistent HCMV detection in blood, saliva, and urine throughout the first years of life [4]. Nevertheless, only 10% to 15% of infected fetuses will develop severe disease involving the brain [5]. Abnormalities of the infected fetal brain range from mild lesions including calcifications, vasculitis, or sub-ependymal cysts that carry an uncertain prognosis to severe ones including microcephaly, abnormal gyration, and white matter necrosis, consistently correlated with a dismal prognosis [6]. Therefore, the detection of brain anomalies in an infected fetus often leads to the termination of pregnancy (TOP).

The pathogenesis of severe HCMV infection in the fetal brain is not well understood. The respective role of viral replication and immune response in the pathogenesis of these severe brain injuries is not well known [7]. However, knowledge of these respective roles could be important for both prenatal and postnatal management of these infections. The objective of this study was to investigate in situ virological and immunological correlates in human fetal brain samples either HCMV-infected with severe or moderate lesions or uninfected.

## 2. Materials and Methods

### 2.1. Fetal Brain Samples

Cases were obtained from HCMV-infected fetuses following maternal primary infection in the first trimester, presenting with cerebral abnormalities detected by ultrasound examination. TOP were performed between 23 and 28 weeks of gestation. Brain samples’ lesions were classified as severe (group A) or as moderate (group B) on the basis of macroscopic and microscopic data analyzed by 2 pathologists blindly and independently. Brain samples were categorized 1) as severely affected (group A) in cases with severe cerebral lesions including microcephaly (<5th percentile), ventriculomegaly, necrosis of the white matter, gyration abnormalities, and polymicrogyria or 2) as moderately affected (group B) in cases with mild abnormalities including cerebral vasculitis, intra-ventricular hemorrhage, germinolysis, and ependymitis. Table S1 reports the macroscopic and microscopic cerebral anomalies detected at postmortem examination in cases and controls. Controls were brain samples obtained from non-infected fetuses matched for gestational age and terminated for severe anomalies other than cerebral abnormalities.

The samples consisted of 26 fetal brains embedded in paraffin (13 severely affected, 8 moderately affected, and 5 controls) collected between 2000 and 2013.

### 2.2. Immunohistochemistry

Brain samples were fixed in formol zinc special (Microm Microtech, Francheville, France), then embedded in paraffin. Coronal sections of 8 µm, performed using the RM2145microtome (Leica^®^, Nanterre, France), included the cortical zone, the white matter, the ventricular zone, and the germinative zone (Figure S1). Antibodies and isotypes are detailed in Table S2. CD8/CD20/MUM-1/NKp46/CD68/PD-1/PD-L1/LAG-3/Tim-3 immunostaining was made at the immunology platform of the European Hospital Georges Pompidou, Paris, using the automate Benchmark XT^®^, Ventana Medical System (Roche diagnostic, Mannhein, Germany). HCMV (IE) staining was performed in the pathology laboratory of Necker hospital using a Leica Bond Max automated IHC/ISH staining instrument (Leica Biosystems, Wetzlar, Germany). NKG2C staining was done manually. In all cases, staining was realized with DAB-chromogen (Dako, Glostrup, Denmark).

The NanoZoomer 2.0 HT Digital slide scanner (Hamamastu photonics^®^, Massy, France) was used to scan immuno-labeled slides.

### 2.3. Quantification of Immunostained Cells (Mean Number of Stained Cells per Tissue Area)

The number of stained cells (CD8/CD20/CD68/MUM-1/PD-1 and LAG-3) per tissue areas was calculated using the computerized image analysis system Developer XD (Definiens Company^®^, Munich, Germany). With this method, each tissue area is divided into tiles consisting of 0.8 mm slices, and the mean density is the ratio of the number of immuno-labeled cells over the tiles’ surface.

The staining density of HCMV- and NKp46-positive cells could not be quantified by image analysis, because both cell types were present in small quantities, and staining of NKp46-positive cells was too weak. For these 2 antibodies, labeled cells present on the slide were counted manually, and the tissue surface was extrapolated from that calculated by image analysis of a consecutive section of the same brain sample.

The density of NKG2C-positive cells could not be calculated because of too strong a background signal for quantitative imaging and too high a number of positive cells for manual quantification. PD-L1 density could not be calculated because positive cells showed low staining intensity and aggregated in clusters, making both manual count and quantitative imaging impossible.

### 2.4. Statistical Analysis

Fischer test and correlation tests were done on Excel version 2010. GraphPad Prism version 6.0 was used to perform Kruskal–Wallis test with Dunn’s Multiple Comparison’s Test and Wilcoxon–Mann–Whitney test and to determine *p*-value and Standard Error of the Mean (SEM). Only *p* values <0.05 were considered significant. Principal component analysis was performed to analyze sets of correlated densities. PCA is a standard method to transform a set of possibly correlated variables into a set of orthogonal (i.e., non-correlated) variables. A visual analogy is to redefine a 3D XYZ scale so that an ellipsoid-shaped set of correlated points in space is transformed into a sphere of uncorrelated coordinates. Therefore, the transformation of a set of x variables yields a set of x non-correlated new variables which are a linear combination of the original ones. We used the first two components (those with most variance, so that the data would spread out) to graphically represent multidimensional data on a bidimensional scatterplot.

All analyses were performed using the R software version 361, Foundation for statistical computing, Vienna, Austria.

### 2.5. Ethical Statement

All procedures were approved by the ethics committee (Agence de Biomedecine, approval: PFS 15-009), and written parental consent was signed for participation in this research.

## 3. Results

### 3.1. Immune Cells and HCMV-Infected Cells

**Figure 1:**
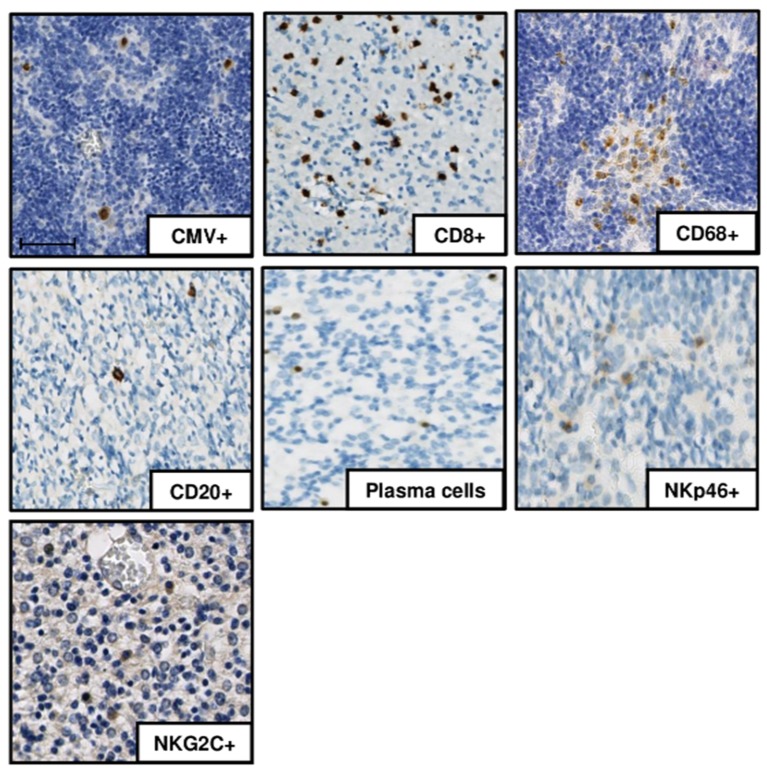
Immunohistochemistry results in one of the severely affected brain samples (case 7). DAB-chromogen was used, and slides were scanned with a NanoZoomer 2.0 HT Digital slide scanner. human cytomegalovirus (HCMV)-positive cells, CD8^+^ cells, CD20^+^ cells, plasma cells, NK cells (NKp46^+^, NKG2C^+^), and macrophages (CD68^+^ cells) are presented. Scale bar 50 µm

Detection: HCMV-positive cells as well as adaptive immune cells (CD8^+^, CD20^+^, plasma cells) and macrophages (CD68^+^) were detected in all infected cases (Figure 1). NKp46-positive cells were detected in all severe cases but in only four of seven moderately affected ones. HCMV-positive cells, plasma cells, NKp46-positive cells, and macrophages were not detected in the controls. The expansion of an NKG2C-positive clone is characteristic of CMV infection [8,9,10,11]. NKG2C immunostaining was negative in the controls, whereas NKG2C receptor staining was identified in 8 of the 11 severe cases and in 1 of the 7 moderately affected brains, (*p* = 0.049, OR 13.2 (IC95% [1.002; 819.6]).

**Figure 2:**
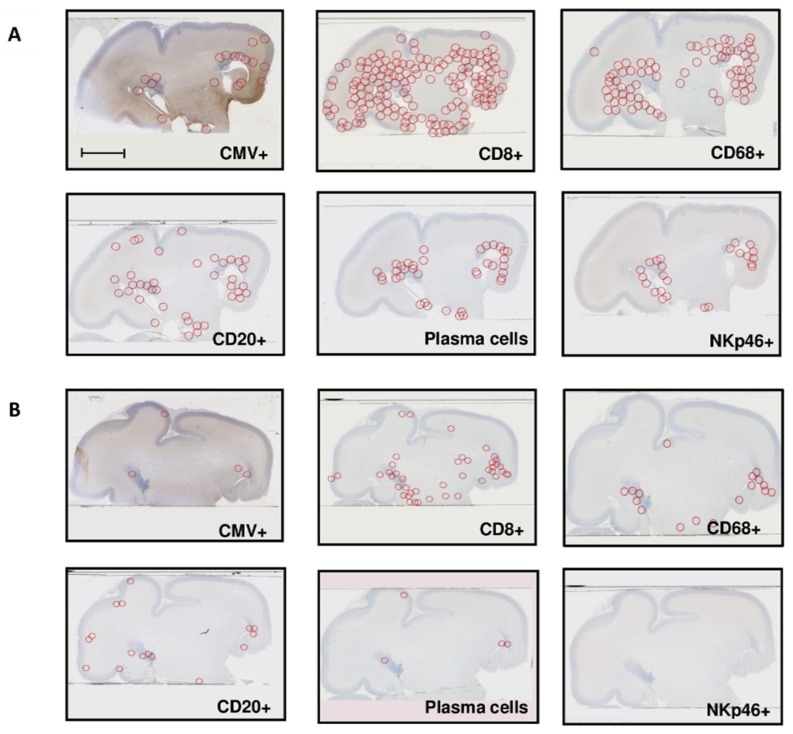
Repartition of HCMV-positive cells and immune cells (**A**) in one severely affected fetal brain sample (group A), successive cuts (case 8), and (**B**) in one moderately affected fetal brain sample (group B), successive cuts (case 17). Scale bar 35 mm.

Localization: immunostained cells were preferentially located in the peri-ventricular and germinative areas in all cases, irrespective of the severity of the cerebral lesions (Figure 2A,B). CD8^+^ cells and macrophages were also found diffused throughout the whole cerebral parenchyma in the most severely affected brains (Figure 2A).

**Figure 3:**
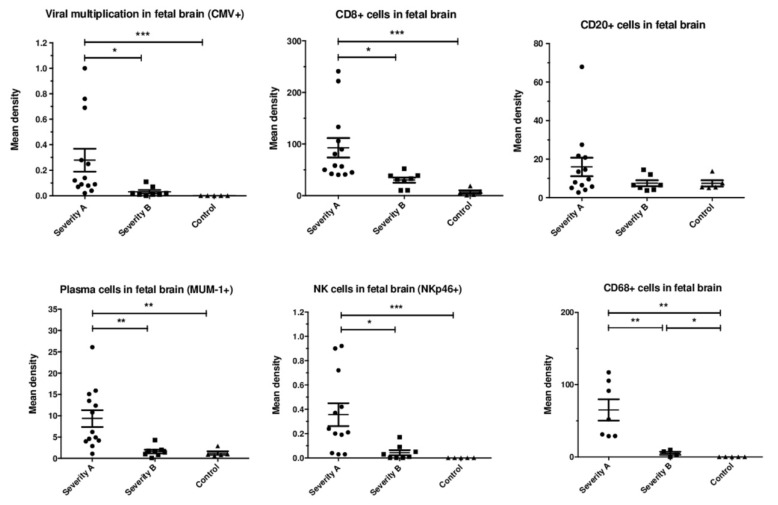
Mean immunostaining densities of HCMV-positive cells and immune cells according to the severity of the brain lesions: severe (group A, *N* = 13), moderate (group B, *N* = 8), and controls (*N* = 5); * *p* < 0.05, ** *p* < 0.01, *** *p* <0.001. Kruskal–Wallis test was used with Dunn’s Multiple Comparison’s Test and Mann–Whitney test.

Quantification: Viral infection (density of HCMV-positive cells) was significantly higher in severely affected brains than in moderately affected ones (*p* = 0.03) (Figure 3). The staining densities of immune cells were higher in severe than in moderate cases and controls for CD8^+^ cells (*p* = 0.014, *p* = 0.0002, respectively), plasma cells (*p* = 0.007, *p* = 0.0032, respectively), NKp46^+^ cells (*p* = 0.02, *p* = 0.0007, respectively) and macrophages (*p* = 0.0061, *p* = 0.0025, respectively). However, they were similar for CD20^+^ cells (*p* = 0.6, *p* = 0.93, respectively). There was no significant difference in the staining densities of immune cells between moderately affected brains and controls, except for CD68^+^ (*p* = 0.048).

### 3.2. Immune Checkpoint Receptors PD-1, PD-L1, LAG-3, and Tim-3

The coexistence of high HCMV densities and high immune cells densities in severely affected brains raises the question of the functionality of these immune cells in the control of viral replication. Exhausted immune cells are expected to express high levels of immune checkpoint receptors such as PD-1, Lag-3, and Tim-3.

**Figure 4:**
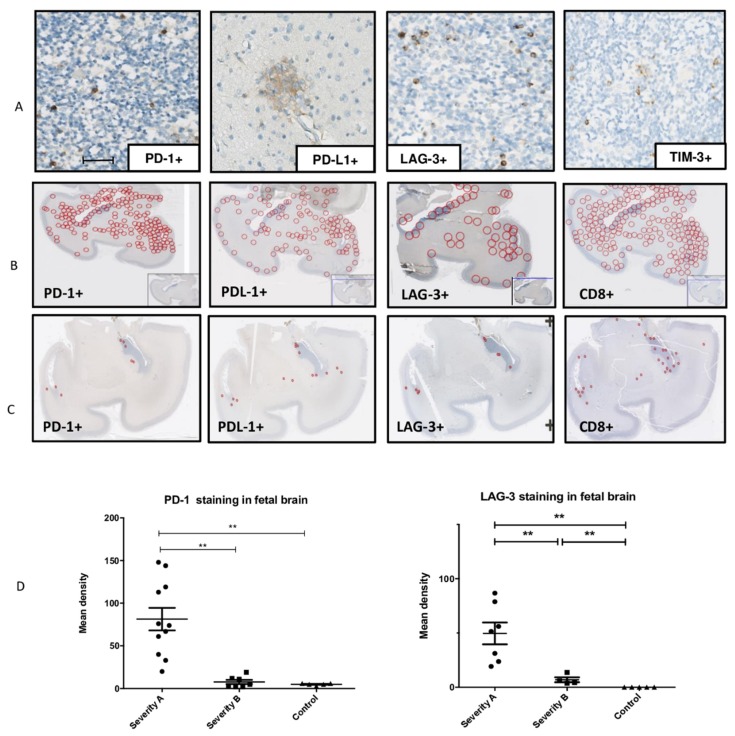
Detection, localization, and quantification of PD-1^+^ cells, PD-L1^+^ cells, LAG-3^+^ cells, and TIM-3^+^ cells in infected fetal brains. (**A**) Detection of PD-1, PDL-1, LAG-3, and TIM-3 immunostaining in one of the severely affected brains (case 7), scale bar 50 µm. (**B**) Localization of PD-1, PDL-1, LAG-3, and CD8 immunostaining in one of the severely affected brain samples and (**C**) in a moderate affected brain sample. (**D**) Mean immunostaining densities of PD-1 and LAG-3 according to the severity of the brain lesions: severe (group A), moderate (group B), and controls; ** *p* < 0.01, *** *p* < 0.001. Kruskal–Wallis test was used with Dunn’s Multiple Comparison’s Test and Mann–Whitney test.

Detection: PD-1, PDL-1, the receptor of PD-1, and LAG-3 were detected in all infected brains but not in controls (Figure 4A). Tim-3 was expressed in both infected brains and controls, probably because Tim3 has been reported to be expressed in normal neuronal cells [12]. Therefore, we did not analyze Tim-3 expression any further.

Localization: The localization of PD-1- and LAG-3-positive cells overlapped with that of CD8^+^ cells: the cells were preferentially localized in the periventricular zones but also widely spread within the cerebral parenchyma in the severe cases (Figure 4 B). PD-L1 immunostaining was found in the same area as that of PD-1, mainly in the periventricular and cortical zones and in the white matter at large (Figure 4B,C).

Quantification: The mean densities of PD-1 and LAG-3 were significantly higher in severe cases than in moderate or controls cases (*p* = 0.001, *p* = 0.004 and *p* = 0.0071, *p* = 0.0025, respectively) (Figure 4D).

### 3.3. Principal Component Analysis between HCMV, Adaptive or Innate Responses, and Markers of Cellular Exhaustion

**Figure 5:**
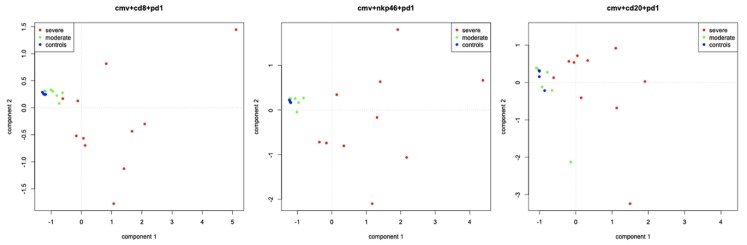
Principal component analysis of CD8, or NKp46, or CD20 and viral multiplication (HCMV) and PD-1 in brain lesions according to cerebral severity (A (severe)/B (moderate)/controls). Severe cases are represented by red dots, moderate cases by yellow dots, and controls by blue dots.

A principal component analysis was performed to bring out density patterns of HCMV-positive cells, adaptive responses (CD8^+^ cells and plasma cells/MUM-1) and innate response (NK cells and macrophage/CD68), and markers of cellular exhaustion (PD-1/LAG-3) according to cerebral severity (groups A, B, or control). A strong link was found between brain lesion severity (severe, moderate, and not-infected) and 1) expression of HCMV/CD8/PD-1 and 2) expression of HCMV/NKP46/PD-1 (Figure 5). In contrast, the expression of HCMV/CD8/CD20/MUM1, HCMV/CD8/CD20/MUM1/PD-1/LAG-3, HCMV/CD20/PD-1 (Figure 5), HCMV/MUM-1/PD-1, HCMV/CD20/LAG-3, HCMV/MUM-1/LAG-3, HCMV/NKp46/CD68/PD-1/LAG-3, HCMV/NKp46/PD-1, HCMV/CD68/PD-1, HCMV/NKp46/LAG-3, and HCMV/CD68/LAG-3 demonstrated a weak or inexistent link with brain lesions’ severity.

## 4. Discussion

Our results demonstrate a significant association between severe fetal brain damage and high levels of innate immune cells in the brain. This is the first study to investigate NK cells’ presence in HCMV-infected human fetal brains. NKp46^+^ cytotoxic cells [13,14] were found in all infected brains but not in controls, and the labelling density was significantly higher in severe than in moderate cases (*p* = 0.02). HCMV infection has been associated with an expansion of NKG2C^+^ cells in both postnatal and prenatal infections [15,16]. NKG2C^+^ cells were found more often in severely affected than in moderately affected brains (*p* = 0.049). This is consistent with previous results showing more frequent and higher expansion of circulating NKG2C^+^ cells in symptomatic neonates compared to asymptomatic ones [16]. Macrophages’ density was also significantly higher in severely affected than in moderately affected brains, in consistency with experimental models of CNS infection demonstrating that blood proinflammatory monocytes are recruited into the brain [17,18,19]. In the mouse model of West Nile Virus encephalitis, depletion of inflammatory macrophages gave conflicting results, with increased levels of local virus replication and decreased survival in one study [18] but increased survival in another study [17]. In the model of murine CMV (mCMV) encephalitis of newborn mice, recruitment of inflammatory macrophages led to inflammation and altered brain development [19].

CD20^+^ lymphocytes were recruited in infected brains, with a trend towards higher densities of CD20^+^ immune labeling in severely affected brains compared to moderately affected ones and controls, but this difference was not significant, as reported previously [20]. Plasma cells were detected in infected brains as previously reported [21], and their density was significantly higher in severely affected than in moderately affected brains. The role of humoral immune responses driven by B-lymphocyte-lineage cells in the brain has been reported for different viral infections. In the mCMV model, infection triggers accumulation and persistence of plasma cells within the brain, leading to the production of antibodies with a significant role in controlling the virus [22]. Whether plasma cells in human fetal infected brains produce HCMV antibodies is not known.

CD8^+^ T cells play a major role for the clearance of the virus in postnatal human infection and in the mCMV model. In our study, the level of CD8^+^ cells was significantly higher in severely affected than in moderately affected or controls brains (*p* = 0.014, *p* = 0.0002). This association between the severity of brain lesions and the accumulation of CD8^+^ cells in the brain has been reported in other two histological studies in human fetal brains [20,21]. This could suggest that cytotoxic T cell responses may play a role in the neurological damage resulting from infection. However, although the severely affected brains were those with the highest levels of CD8^+^ cell infiltration, they were also those with the highest levels of HCMV infection (*p* = 0.03). This apparent paradox of concomitant high levels of HCMV infection and high numbers of CD8^+^ T cells as a risk factor for severe brain lesions prompted us to measure the expression of the immune checkpoint receptors PD-1/PD-L1 and LAG-3. These receptors have a crucial role in regulating the immune responses by inhibiting T cell receptor signaling and cytokine production [23] and have become markers of T cell unresponsiveness or exhaustion. The development of CD8^+^ T cell exhaustion with high PD-1 and LAG-3 expression has been associated with chronic infections such as HIV infection or HBV infection [24,25]. We found PD-1- and LAG-3.expressing cells in HCMV-infected brains but not in controls. PD-1/PDL-1- and LAG-3-expressing cells co-localized with CD8^+^ cells and were significantly more expressed in severely affected brains than in moderately affected ones (*p* = 0.001 and *p* = 0.008, respectively). Moreover, a strong link between the combined expression of HCMV/CD8/PD1 as well as the one of HCMV/NKp46/PD1 and the severity of brain lesions was found with component analysis. This is consistent with the study by Coleman et al. reporting that during chronic HIV infection, PD-1 expression was associated with the presence of CD8^+^ cells and also, albeit less frequently, with NK cells [26].

The high expression of immune checkpoint receptors could reflect immune exhaustion of T cells in infected fetal brains. Looking at the literature, two studies give credibility to this hypothesis. First, a study has reported blood T cell exhaustion in HCMV-infected newborns with high PD-1 expression and paucifunctional responses to viral antigens restored by PD-1 blockage [27]. Second, in the mice model of (Herpes simplex virus) HSV encephalitis, brain-resident T cells of the ependyma were shown to coexpress immune checkpoint receptors and ex vivo paucifunctionality, allowing persistent infection in this region [28]. Moreover, in HIV infection, a recent study reported that a higher proportion of differentiated CD8^+^ T cells and an increased PD1 expression were associated with higher HIV reservoir (DNA level) one year after treatment of the primary infection [29]. However, the high expression of immune checkpoint receptors should be interpreted with caution, because some investigators have suggested that during infection, this expression was not necessarily equal to exhaustion and could, on the contrary, be related to T cell activation. Indeed, Gabrielli et al. reported the presence of granzyme B-positive cells surrounding cytomegalic cells in a severely infected fetal brain, suggesting that some brain lymphocytes (CD8^+^, NK cells) were activated and potentially led to immune-mediated toxicity [20]. The functionality of brain lymphocytes should be studied to better understand these apparent contradictions. For example, new technologies like the single-cell assessment of cytotoxic protein expression could be used; however, this would imply using fresh brain biopsies, which are difficult to obtain. Moreover, the investigation of the correlation between blood immunological profiles (expression of CD8 and immune checkpoint receptors) and brain lesions should be encouraged, since it might help identifying surrogate neurological prognosis markers.

## 5. Conclusions

These findings show that the expansion of total NK cells and their subset NKG2C^+^ cells is promoted in HCMV-infected fetal brains, as is the recruitment of macrophages in the brain. However, innate responses through NK cells and macrophages do not seem sufficient to mitigate brain viral multiplication, at least in severe cases. Whether innate cell responses could participate in neuropathology, as demonstrated in animal models of encephalitis, remains unknown. Furthermore, our study suggests that effective T cell-mediated control could be impaired in cases with high viral multiplication in the brain. If this hypothesis is correct, brain damage could therefore be directly related to the loss of control of the virus replication and to HCMV direct effect on neuronal cells. These results plead for prompt antiviral treatment to jugulate or at least mitigate viral multiplication as early as possible in the course of fetal infection. This could help to reduce the effect of a sequence of high viral loads potentially triggering deregulated immune responses with possible immune cells exhaustion, thus responsible for increased HCMV replication and cytopathic effects on brain tissue.

## Supplementary Materials

The following are available online at https://www.mdpi.com/2076-2607/8/2/176/s1, Table S1: Macroscopic and microscopic cerebral abnormalities at post-mortem examination of infected brains samples; Table S2: Antibodies used for immunohistochemistry; Figure S1:Coronal sections for immunohistochemistry included the cortical zone, the white matter, the ventricular zone and the germinative zone.
