# Supplementary material for: Adaptive and Innate Immune Cells in Fetal Human Cytomegalovirus-Infected Brains
Source: Microorganisms. 2020 Jan 25;8(2):176. doi: 10.3390/microorganisms8020176 (PMC7074756; doi:10.3390/microorganisms8020176)

Supplementary Data "Adaptive and Innate Immune Cells in Fetal Human Cytomegalovirus-Infected Brains"

Sellier Y et al

Table S1. Macroscopic and microscopic cerebral abnormalities at post-mortem examination of infected brains samples

Fetal pathology	Cases	Maternal age	Weeks at TOP	Microcephaly < 5th Percentile	Necrosis	Calcifications	Ventriculomegaly	Polymicrogyria	Minor brain abnormalities	Cerebral severity
CMV infection	1	38	23	1	0	0	1	1	1	A
CMV infection	2	35	28	0	0	0	0	0	1	B
CMV infection	3	34	25	1	1	1	1	0	1	A
CMV infection	4	28	24	1	1	1	0	0	1	A
CMV infection	5	31	23	1	1	0	0	1	1	A
CMV infection	6	27	27	0	0	0	0	0	1	B
CMV infection	7	33	23	0	1	0	0	1	1	A
CMV infection	8	26	28	1	0	1	0	0	1	A
CMV infection	9	34	24	Unknown	1	0	1	1	1	A
CMV infection	10	28	25	0	1	1	1	0	1	A
CMV infection	11	31	23	0	0	0	0	0	1	B
CMV infection	12	27	23	0	0	0	1	1	1	A
CMV infection	13	31	28	Unknown	0	0	1	1	1	A
CMV infection	14	31	25	1	1	1	1	1	1	A
CMV infection	15	31	28	0	0	0	0	0	1	B
CMV infection	16	37	27	1	1	0	1	1	1	A
CMV infection	17	28	27	0	0	0	0	0	1	B
CMV infection	18	31	23	0	0	0	1	0	1	A
CMV infection	19	27	24	0	0	0	0	0	1	B
CMV infection	20	29	24	0	0	0	0	0	1	B
CMV infection	21	36	28	0	0	0	0	0	1	B
Renal failure	22	30	26	0	0	0	0	0	0	control
Heart failure	23	34	24	0	0	0	0	0	0	control
Maternal psychiatric disorder	24	32	27	0	0	0	0	0	0	control
Anamnios, chorioamnionitis, premature rupture of membranes	25	38	23	0	0	0	0	0	0	control
Heart disorder, intra uterine growth retardation omphalocele	26	35	28	0	0	0	0	0	0	control

0= absence; 1= presence; TOP= Termination of pregnancy

Table S2. Antibodies used for immunohistochemistry

Antibodies	Clone	Species	Isotype	Dilution	Source	Target
CD8	c8/144B	mouse monoclonal	Ig G1	1/50	Dako, Glostrup, Denmark	CD8 T lymphocytes
CD20	L26	mouse monoclonal	Ig G2a	ready to use	VentanaMedical System	CD20 B lymphocytes
MUM-1 (Multiple Myeloma Oncogene-1)	MUM-1	mouse monoclonal	Ig G1	1/50	Dako	Plasma cells
NKp46	195314	mouse monoclonal	Ig G2B	1/100	R&D sytems, Bio-techne, Vauban, France	Natural killer (NK), receptor cytotoxic activator
IE	E13	mouse monoclonal	IgG1k	1/50	Argene, Biomérieux, Marcy l'Etoile, France	Early antigen specific to HCMV
PD-1 (Programmed Death-1)	NAT105	mouse monoclonal	Ig G1	ready to use	Cell Marque, Emergo Europe, The Hague, Netherlands	PD-1 on immune cells
PD-L1 (Programmed Death-Ligand 1)	E1L3N	rabbit monoclonal	Rabbit IgG	1/100	CellSignalingtechnology, Ozyme, Saint Quentin en Yveline, France	PD-L1 (infected cells)
NKG2C	aa1-30	rabbit	polyclonal	1/100	LifeSpan Biosciences, Clinisciences, Nanterre, France	Human NK receptor activator
LAG-3	17B4	mouse monoclonal	Ig G1	1/2000	ABCAM	Lymphocyte (activation gene 3)
TIM-3	D5D5R	Rabbit monoclonal	Rabbit IgG	1/400	Cell Signaling	TIMP family
CD68	Kp1	Mouse monoclonal	IgG1k	1/3000	Agilent Dako	Human macrophage

Figure S1: Coronal sections for immunohistochemistry included the cortical zone, the white matter, the ventricular zone, and the germinative zone

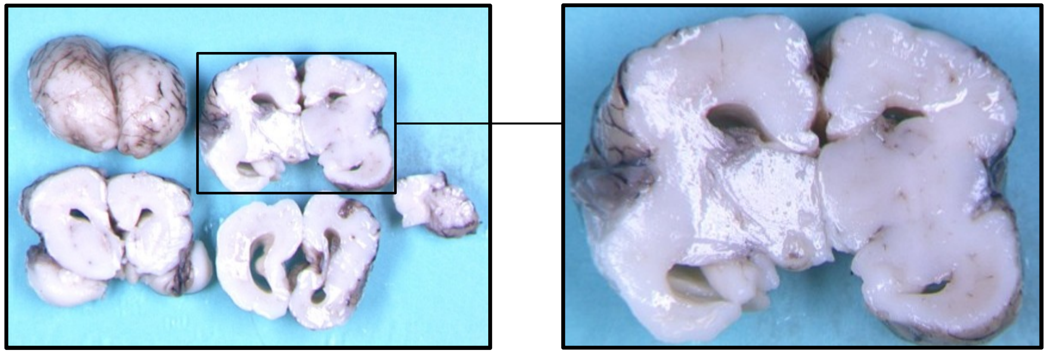

Supplement: Supplementary file 1 [file microorganisms-08-00176-s001.pdf]
